# Food Processing Knowledge in Relation to Healthiness and Self‐Reported Understanding of the Health Star Rating in Australia

**DOI:** 10.1002/hpja.70235

**Published:** 2026-09-03

**Authors:** Jacqueline Penney, Tailane Scapin, Adrian J. Cameron, Christine M. White, David Hammond, Clara Gomez‐Donoso

**Affiliations:** ^1^ Tasmanian School of Medicine University of Tasmania Hobart Australia; ^2^ Global Centre for Preventive Health and Nutrition, Institute for Health Transformation, Deakin University Geelong Australia; ^3^ School of Public Health Sciences University of Waterloo Waterloo Canada; ^4^ NUTRALiSS Research Group, Faculty of Health Sciences Universitat Oberta de Catalunya Barcelona Spain

**Keywords:** food policy, food processing, front‐of‐pack labelling, healthiness knowledge

## Abstract

**Issue Addressed:**

This study aimed to examine Australians' food processing knowledge in relation to food healthiness and self‐reported understanding of the Health Star Rating (HSR), their association, and sociodemographic differences.

**Methods:**

An online survey was completed by 3760 adults in Australia as part of the 2021 International Food Policy Study. Participants rated the healthiness of foods across four categories (grains, meat, dairy, fish), with three products presented per category representing increasing levels of processing. Food processing knowledge (FoodProK) scores were based on the alignment between healthiness ratings and processing level. Participants also self‐reported their ease of understanding the HSR. Multivariable regression analyses were conducted.

**Results:**

The mean FoodProK was 4.8 (of 8 points); 63% of respondents found the HSR label very easy/easy to understand. FoodProK was positively associated with HSR understanding. Females and older respondents had a higher FoodProK, while those aged 60+ had lower HSR understanding. Respondents who identified as Aboriginal and/or Torres Strait Islander had a lower FoodProK and HSR understanding. Following dietary restrictions and not being involved in food shopping were associated with lower FoodProK. Higher self‐reported nutrition knowledge, diet healthiness and income adequacy were associated with higher FoodProK and/or HSR understanding.

**Conclusions:**

The study found a moderate FoodProK, a generally adequate HSR understanding, and a positive association between self‐reported HSR understanding and food processing knowledge in relation to food healthiness, although notable sociodemographic inequities were observed.

**So What:**

Tailored communication strategies may help improve understanding of the HSR and food processing in relation to healthiness across population groups.

## 1 Introduction

Dietary risk factors are one of the leading modifiable contributors to the global burden of obesity and non‐communicable diseases [1]. In Australia, unhealthy dietary behaviours are increasingly prevalent, with discretionary or unhealthy food (i.e., energy dense, high in added sugar, saturated fat and sodium and low in beneficial nutrients) accounting for over a third of daily energy intake [2, 3]. In contrast, the consumption of healthy foods like whole grains, fruit and vegetables falls well below Australian Dietary Guidelines recommendations [2, 3]. While dietary risk factors can be modified, they are complex in nature and influenced by a multitude of interpersonal, biological, social, cultural, economic and environmental factors [4].

Contemporary food environments have been referred to as ‘obesogenic’, encouraging the consumption of unhealthy diets through the excessive promotion, availability, and affordability of unhealthy food and beverages [5, 6], especially in the most socioeconomically deprived areas [7]. As a result, food policies including front‐of‐pack nutrition labelling (FOPNL) schemes have been proposed and implemented to support healthy food environments and assist individuals to identify healthier food options [8, 9]. Nutrient‐based food classification schemes have been the most common systems used to identify healthier options in recent decades, including the voluntary Health Star Rating (HSR) implemented in Australia and New Zealand since 2014 [10]. However, with an increasingly industrialised food system, new food classification systems have been developed that classify foods based on other characteristics such as the extent, nature and purpose of food processing [11, 12]. The NOVA system is the most widely utilised and endorsed food processing classification system [12], categorising foods into four groups: unprocessed or minimally processed foods, processed culinary ingredients, processed foods, and ultra‐processed foods (UPF).

Evidence shows that diets high in UPF are associated with increased risk of non‐communicable chronic diseases [13]. Although many UPF are characterised by poor nutrient profiles, some receive favourable ratings under interpretative nutrient profiling systems like the HSR [14]. This highlights that nutritional quality and food processing are related, but conceptually distinct dimensions of food healthiness. Consequently, it has been proposed to incorporate information on the level of processing into nutrient‐based FOPNL schemes [15, 16]. In Australia, the HSR system is currently implemented as a voluntary FOPNL scheme, although a public consultation is underway regarding potential mandatory adoption [17]. As part of this consultation, broader revisions to the system are being considered, with some public health groups supporting a review of the HSR nutrient profiling algorithm to account for ultra‐processing [16], reflecting growing interest in the role of food processing in nutrition policy frameworks.

Even though food processing level is increasingly incorporated into dietary guidelines and is being considered as a potential complement to existing FOPNL schemes [15, 16], evidence regarding individuals' understanding of food processing in relation to food healthiness remains limited, particularly in countries where this dimension has not yet been incorporated into food policy strategies [18, 19, 20, 21, 22]. Recent evidence from Australia highlights concern and confusion around food processing terminology, with a general understanding of key UPF characteristics but frequent misclassification [21, 22]. On the other hand, the latest food Standards Australia New Zealand (FSANZ) evidence from 2025 [23] shows that awareness of the nutrient‐based HSR is very high in Australia, with the vast majority reporting at least some knowledge of it and demonstrating an adequate understanding.

Nutrition and food literacy play a key role in understanding dietary information, identifying healthy dietary options, and guiding food purchasing decisions [24, 25]. Previous research has assessed the use and understanding of different nutrient‐based FOPNL systems [26, 27] and food processing knowledge [20, 21, 22, 28]. However, no studies have evaluated the association between both measures in Australia. Understanding how people interpret both nutrient and processing dimensions of food healthiness is increasingly relevant as dietary guidelines, food policy frameworks, food communication and public discourse are evolving in this direction. Additionally, identifying potential inequities across population groups can help tailor public health programs and communication strategies aimed at supporting a more equitable impact of nutrient‐based labelling policies like the HSR and improving understanding of food processing. This study extends previous research by assessing the relationship between HSR understanding and food processing knowledge as well as examining differences in these measures by sociodemographic characteristics, body mass index (BMI), and diet‐related behaviours among Australian adults.

## 2 Methods

### 2.1 Study Design and Participants

Data from Australian participants in the 2021 wave of the International Food Policy Study (IFPS) were analysed. The IFPS is an annual, web‐based cross‐sectional survey conducted in Australia, Canada, Mexico, United Kingdom and United States that assesses dietary patterns and policy‐relevant behaviours. The survey enables evaluation of the impact of national‐level changes in food policy on diet‐related knowledge, attitudes, and behaviours. The Australian survey was conducted in English, with questions and terminology adapted to be country specific. Recruitment was conducted by Nielsen Consumer Insights Global Panel and their partner panels using probability and non‐probability sampling techniques. Invitations to participate were emailed to randomly selected potentially eligible panellists after targeting for demographics. Potential respondents were provided with information about the study and provided consent prior to participating. Eligibility criteria included being 18+ years and an Australian resident, and considered quotas for age, sex and education. Participants were remunerated in accordance with their panel's incentive structure (e.g., points‐based or monetary rewards, chances to win prizes). For further detail on the research methodology, please refer to the IFPS Technical Report [29]. The study received ethics clearance through a University of Waterloo Research Ethics Committee (REB #30829).

### 2.2 Food Processing Knowledge in Relation to Food Healthiness (FoodProK)

The FoodProK score assessed food processing knowledge in relation to food healthiness by evaluating whether participants correctly identified foods as healthier or less healthy according to their level of processing. The extent to which healthiness ratings aligned with products' processing levels was used as a measure of implicit understanding of the relationship between food processing and food healthiness. The tool was developed as a proxy for overall nutrition knowledge, with the degree of processing considered a sufficiently comprehensive indicator of food healthiness and suitable for cross‐country comparisons, unlike context‐specific measures based on national dietary recommendations. Its content validity was confirmed with registered dietitian nutritionists in Canada [30].

**FIGURE 1:**
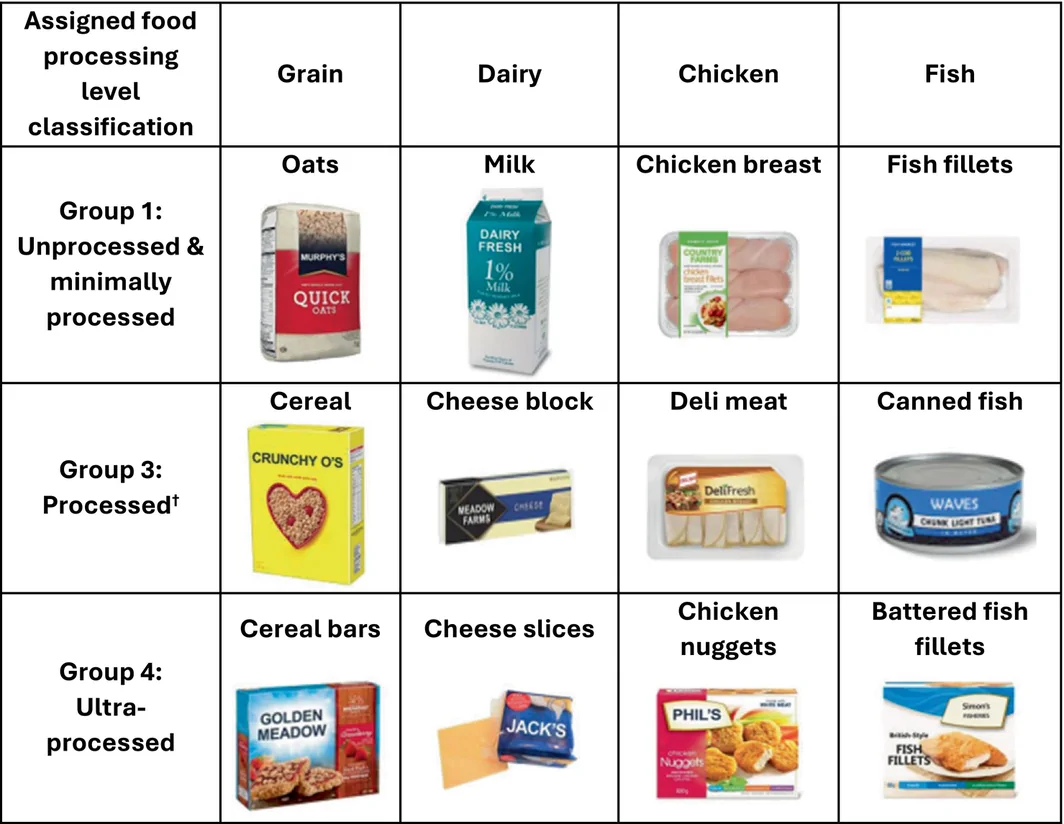
Food products* included in the Food Processing Knowledge (FoodProK) score by assigned food processing level classification. International Food Policy Study, Australia, 2021. *Respondents were shown the above product images accompanied by nutrition facts panels and ingredient lists (when applicable). †The cereal and cheese block products contained ingredient(s) characteristic of ultra‐processed products.

As part of the IFPS survey, respondents were shown images of 12 food products from four food categories (meat, dairy, grains, fish) with three products within each category (Figure 1) intended to represent differing food processing levels based on three of the NOVA food classification groups: group 1—unprocessed or minimally processed; group 3—processed; or group 4—ultra‐processed (note, no group 2 products—culinary ingredients—were included). However, as further described in the study limitations, there were some challenges in selecting the products intended to reflect group 3 processed foods in the dairy and grain categories (i.e., the cheese block and cereal products shown in the FoodProK task contained ingredient(s) characteristic of UPF). Compared with the original FoodProK task developed by Bhawra and colleagues [30], the FoodProK in the 2021 IFPS survey included products from a fish category instead of a fruit category.

Respondents were tasked with rating the healthiness of each of the 12 food products, one at a time, on a scale from 0 (not at all healthy) to 10 (extremely healthy). Brand names on the product packaging were replaced with fictional names to minimise bias associated with familiarity. Nutrition fact tables and ingredients lists were shown beside packaged food products (when applicable), as per in a real shopping experience, to assist respondents in rating product healthiness. Respondents were assigned a maximum FoodProK score of 2 for each category if the healthiness of food products was correctly rated according to their intended level of food processing (i.e., the unprocessed/minimally processed product was rated healthier than the processed product, and both were rated healthier than the ultra‐processed product), a score of 1 if only one item was in the correct relative position, or a score of 0 if none were correctly rated. Scores for the four categories (ranging from 0 to 2) were then added together to generate the overall FoodProK score (ranging from 0 to 8). Higher scores indicated a higher alignment between food healthiness and processing level, suggesting that processing level may serve as an implicit heuristic when participants assess food healthiness. Respondents who had missing data (skipped or selected ‘refuse to answer’ for one or more food items) or selected ‘don't know’ for more than 50% of the items, were excluded from the FoodProK scores; otherwise ‘don't know’ responses were considered as incorrect (i.e., category score was 0).

### 2.3 Modified FoodProK

Given the challenges associated with the overlap between NOVA group 3 (processed) and 4 (ultra‐processed) classifications among products shown for the dairy and grain categories, and since UPF are the main NOVA group of concern for public health [11, 12, 13], a modified FoodProK score was also calculated to conduct sensitivity analyses. The modified FoodProK score was calculated based only on responses for products that represent the lowest (unprocessed/minimally processed) and highest levels of processing (ultra‐processed). For each category, respondents who rated unprocessed and minimally processed (NOVA group 1) items as healthier than UPF items (group 4) were assigned 2 points, without considering responses for processed items (group 3). Total modified FoodProK scores could range up to a maximum of 8 points.

### 2.4 Self‐Reported Understanding of the Health Star Rating (HSR)

To assess self‐reported understanding of the HSR label, respondents were shown a generic HSR image with a 3.5 rating (by itself, not applied to a product) and were asked: “Do you find this information…”, with response options including ‘very hard to understand’, ‘hard to understand’, ‘neither hard nor easy’, ‘easy to understand’ and ‘very easy to understand. For the logistic regression analysis, self‐reported understanding of HSR was examined using a dichotomous variable (0 = very hard/hard/neither, 1 = easy/very easy). Respondents that selected ‘don't know’ or ‘refuse to answer’ were excluded.

### 2.5 Sociodemographic Characteristics

Self‐reported sociodemographic measures included: sex at birth (male, female); age group (18–29, 30–44, 45–59, 60+ years); region (state or territory); education level (low—year 12 or lower, medium—trade certificate or diploma, high—bachelor's degree or above); perceived income adequacy to make ends meet (very difficult/difficult, neither easy nor difficult, easy/very easy); language spoken at home (only English, other language(s)) Indigeneity (Non‐Indigenous, Aboriginal and/or Torres Strait Islander).

### 2.6 Body Mass Index (BMI)

BMI was calculated using self‐reported height and weight and was categorised into five classifications: ≤ 18.5 kg/m^2^, 18.5–24.9 kg/m^2^, 25–29.9 kg/m^2^, ≥ 30.0 kg/m^2^ and missing BMI (including ‘refuse to answer’, ‘don't know’ and implausible extreme values).

### 2.7 Diet‐Related Practices

#### 2.7.1 Food Shopping Role

To determine their food shopping role, respondents were asked “How much of the food shopping do you do in your household?”, with response options including ‘most’, ‘share equally with others’, ‘some, but less than other(s)’, and ‘none’.

#### 2.7.2 Self‐Reported Nutrition Knowledge

Respondents were asked to self‐assess their level of nutrition knowledge by rating their understanding through five response options ranging from ‘not at all knowledgeable’ to ‘extremely knowledgeable’, which were recoded into three analytic categories (not at all/a little knowledgeable, somewhat knowledgeable, very/extremely knowledgeable).

#### 2.7.3 Self‐Reported Healthiness of Diet

To assess the healthiness of diet respondents were asked “In general, how healthy is your overall diet?”, with five response options ranging from ‘poor’ to ‘excellent’ categorised into three groups (poor/fair, good, very good/excellent).

#### 2.7.4 Dietary Restrictions

Respondents were asked to indicate if they followed specific dietary practices, including vegetarian, vegan, pescatarian or following a religious practice for eating.

### 2.8 Analysis

Post‐stratification sample weights were constructed using a raking algorithm with Australian population estimates based on age group, sex, region, ethnicity and education. Weighted descriptive statistics were used to summarise sample characteristics, FoodProK scores and self‐reported understanding of HSR. A multivariable linear regression analysis was conducted to evaluate the relationship between FoodProK score and sociodemographic and diet‐related characteristics. A multivariable logistic regression analysis was conducted to investigate the association between understanding of HSR and sociodemographic and diet‐related characteristics. Multivariable regression analyses were also conducted to examine the association between FoodProK and HSR. Sensitivity analyses were conducted using the modified FoodProK score. Responses of ‘refuse to answer’ and ‘don't know’ for sociodemographic and other covariates (except BMI) were excluded for regression analysis. Analyses were conducted using Stata version 17.

## 3 Results

A sample of 3760 respondents was included in the descriptive analysis after excluding missing data as well as ‘don't know’ and ‘refuse to answer’ responses from the main outcomes of interest: FoodProK score (*n* = 284) and understanding of HSR (*n* = 61). A sub‐sample of 3645 was retained for the multivariable regression analysis after further excluding respondents with missing data for sociodemographic and diet‐related characteristics (except for BMI).

Table 1 shows the weighted characteristics for the study sample. Most respondents only spoke English at home, and 5% identified as Aboriginal and/or Torres Strait Islander. Over a third of respondents were within the ‘18.5–24.9 kg/m^2^’ BMI category, with over 45% in the ‘25–29.9 kg/m^2^’ or ‘≥ 30.0 kg/m^2^’ categories. Over two‐thirds of respondents did most of the food shopping for their household. One fifth of respondents reported they were very/extremely knowledgeable about nutrition. More than two‐thirds rated the healthiness of their diet as good, very good or excellent. Most respondents did not follow dietary restrictions.

**TABLE 1:** Weighted sociodemographic and dietary characteristics.

Sociodemographic and dietary characteristics	Weighted % (*n*)
Sex at birth	
Male	49.3 (1853)
Female	50.7 (1907)
Age group (years)	
18–29	20.6 (776)
30–44	27.2 (1024)
45–59	24.0 (900)
≥ 60	28.2 (1060)
Education level	
Low	41.6 (1563)
Medium	32.0 (1202)
High	26.0 (982)
Not Stated	0.4 (13)
Perceived income adequacy	
Very difficult/difficult	22.5 (845)
Neither	36.0 (1353)
Easy/very easy	40.6 (1528)
Not stated	0.9 (34)
Language spoken at home	
Only English	76.1 (2862)
Other language(s)	23.5 (883)
Not Stated	0.4 (15)
Indigeneity	
Non‐Indigenous	94.4 (3550)
Aboriginal and/or Torres Strait Islander	5.1 (191)
Not stated	0.5 (19)
Region	
New South Wales	32.0 (1205)
Victoria	25.5 (959)
Queensland	20.0 (751)
Western Australia	10.5 (392)
South Australia	7.0 (264)
Tasmania	3.1 (117)
Australian Capital Territory	1.7 (64)
Northern Territory	0.2 (8)
Body Mass Index (BMI) (kg/m^2^)	
< 18.5	3.9 (147)
18.5–24.9	34.3 (1291)
25.0–29.9	25.7 (967)
≥ 30.0	20.4 (765)
Missing	15.7 (590)
Food shopping role	
Most	69.1 (2599)
Shared equally with others	22.3 (837)
Some, but less than others	6.7 (254)
None	1.7 (64)
Not stated	0.2 (6)
Self‐reported nutrition knowledge	
Not at all/a little knowledgeable	39.9 (1499)
Somewhat knowledgeable	39.8 (1498)
Very/extremely knowledgeable	19.6 (736)
Not stated	0.7 (27)
Self‐reported diet healthiness	
Poor/fair	30.7 (1155)
Good	43.1 (1620)
Very good/excellent	25.3 (950)
Not stated	0.9 (35)
Dietary restrictions	
One or more (i.e., vegetarian, vegan, pescatarian, religious practices)	16.0 (605)
None	83.3 (3131)
Not stated	0.7 (24)

*Note:* International Food Policy Study, Australia, 2021 (*n* = 3760).

### 3.1 Self‐Reported HSR Understanding and FoodProK

As shown in Table 2, the majority (63%) of respondents found the HSR easy or very easy to understand. Just over a quarter found the HSR neither hard nor easy to understand, while approximately 11% found it hard or very hard to understand.

**TABLE 2:** Self‐reported understanding of the Health Star Rating (HSR) label.

Self‐reported understanding of the health star rating	Percentage (95% CI)
Very hard to understand	2.5 (2.0, 3.1)
Hard to understand	8.2 (7.3, 9.2)
Neither hard nor easy to understand	26.3 (24.8, 27.9)
Easy to understand	44.1 (42.3, 45.8)
Very easy to understand	19.0 (17.6, 20.4)

*Note:* International Food Policy Study, Australia, 2021 (*n* = 3760).

Table 3 shows the mean FoodProK scores for each of the four food categories (each out of 2 points) and overall (out of 8 points). Scores were highest for the chicken category (1.4) and lowest for dairy (1.0). The mean total FoodProK score was 4.8 out of 8. The modified FoodProK scores, which only considered responses for unprocessed/minimally processed and UPF items but used the same rating approach, were higher than the original FoodProK scores for all categories (individually and overall). Modified FoodProK scores were also highest for the chicken category (1.7) and lowest for dairy (1.2), with a mean total score of 6.0 out of 8.

**TABLE 3:** Food processing knowledge (FoodProK) and modified FoodProK scores by category and total^a^.

Category	Mean FoodProK score (SD)	Mean modified FoodProK score^b^ (SD)
Grain	1.3 (0.6)	1.6 (0.8)
Dairy	1.0 (0.6)	1.2 (0.9)
Chicken	1.4 (0.7)	1.7 (0.7)
Fish	1.2 (0.6)	1.5 (0.9)
Total	4.8 (1.7)	6.0 (2.3)

*Note:* International Food Policy Study, Australia, 2021 (*n* = 3760).

^a^ Mean score (original and modified) per category is out of 2 points; mean total score is out of 8 points.

^b^ Based on unprocessed/minimally processed and ultra‐processed food items only (i.e., the processed food category was excluded).

The mean healthiness ratings (ranging from 0 to 10) for each food product, which were used to derive the FoodProK score, are available in Table S1. Individual product ratings show that NOVA group 1 items like chicken breast (7.9 out of 10) and fish fillets (7.8 out of 10) had the highest mean healthiness ratings, whereas group 4 items like cereal bars (2.6 out of 10) and cheese slices (3.0 out of 10) had the lowest mean healthiness ratings.

As shown in Table 4, there was a positive, moderate association between the FoodProK score and self‐reported HSR understanding, with respondents who perceived the HSR front‐of‐pack label as easy/very easy to understand having significantly higher FoodProK scores compared to those that found it very hard/hard to understand.

**TABLE 4:** Association between the Food Processing Knowledge (FoodProK) score and self‐reported understanding of Health Star Rating (HSR) labelling^a^.

Health star rating self‐reported understanding	Total mean FoodProK^b^	Coefficient (*β*)	95% CI	*p*
Very hard or hard	4.5	Reference		
Neither hard nor easy	4.7	0.19	−0.33, 0.41	0.097
Easy or very easy	**4.9**	**0.44**	**0.24, 0.64**	**< 0.001**

*Note:* International Food Policy Study, Australia, 2021 (*n* = 3645). In bold: Statistically significant (*p* < 0.05).

^a^ Adjusted for sociodemographic characteristics (sex, age, education, perceived income adequacy, language spoken at home, Indigeneity).

^b^ Weighted mean (unadjusted for covariates).

### 3.2 Association Between FoodProK and Sociodemographic and Diet‐Related Characteristics

Results from the linear regression analysis examining differences in FoodProK scores across sociodemographic and diet‐related characteristics are presented in Table 5. Females had significantly higher FoodProK scores than males. Age was also significantly associated with FoodProK scores, with those ≥ 60 years and 45–59 years having significantly higher scores compared to the youngest age group (18–29 years old). Respondents who identified as Aboriginal and/or Torres Strait Islander had significantly lower FoodProK scores compared to Non‐Indigenous people. Respondents with missing BMI data had significantly lower FoodProK scores compared to those with a BMI between 18.5–24.9 kg/m^2^. There were no statistically significant differences in FoodProK scores by education level, perceived income adequacy, language spoken at home, or self‐reported diet healthiness.

**TABLE 5:** Association between the Food Processing Knowledge (FoodProK) score and sociodemographic/diet‐related characteristics.

Variable	Coefficient (*β*)	95% CI	*p*
Sex at birth			
Male	Reference		
Female	**0.27**	**0.15, 0.38**	**< 0.001**
Age group (years)			
18–29	Reference		
30–44	−0.08	−0.27, 0.12	0.431
45–59	**0.31**	**0.12, 0.50**	**0.001**
≥ 60	**0.58**	**0.40, 0.76**	**< 0.001**
Education level			
Low	Reference		
Medium	−0.06	−0.20, 0.07	0.361
High	−0.09	−0.25, 0.07	0.271
Perceived income adequacy			
Very difficult/difficult	Reference		
Neither	−0.09	−0.25, 0.07	0.285
Easy/very easy	0.04	−0.12, 0.19	0.628
Language spoken at home			
Only English	Reference		
Other language(s)	0.07	−0.10, 0.24	0.435
Indigeneity			
Non‐Indigenous	Reference		
Aboriginal and/or Torres Strait Islander	**−0.55**	**−0.89, −0.21**	**0.001**
Body Mass Index (kg/m^2^)			
18.5–24.9	Reference		
< 18.5	−0.29	−0.62, 0.04	0.081
25.0–29.9	0.02	−0.13, 0.16	0.827
≥ 30.0	0.01	−0.15, 0.17	0.908
Missing	**−0.52**	**−0.71, −0.32**	**< 0.001**
Food shopping role			
Most	Reference		
Share equally	−0.04	−0.18, 0.10	0.537
Some, but not as much as others	0.19	−0.08, 0.46	0.162
None	**−0.88**	**−1.40, −0.35**	**0.001**
Self‐reported nutrition knowledge			
Not at all/a little knowledgeable	Reference		
Somewhat knowledgeable	0.13	−0.002, 0.25	0.054
Very/extremely knowledgeable	**0.24**	**0.07, 0.41**	**0.005**
Self‐reported diet healthiness			
Poor	Reference		
Fair/good	−0.03	−0.17, 0.11	0.702
Very good/excellent	−0.12	−0.28, 0.05	0.175
Dietary restrictions			
None	Reference		
One or more (i.e., vegetarian, vegan, pescatarian, religious practices)	**−0.75**	**−0.94, −0.56**	**< 0.001**

*Note:* International Food Policy Study, Australia, 2021 (*n* = 3645). In bold: Statistically significant (*p* < 0.05).

Respondents who did none of the food shopping exhibited significantly lower FoodProK scores than those who did most of the food shopping. Self‐reported nutrition knowledge was also associated with FoodProK scores, with respondents who reported being very/extremely knowledgeable obtaining significantly higher FoodProK scores compared to those not at all/a little knowledgeable. Finally, respondents following one or more dietary practices (vegetarian, vegan, pescatarian and/or religious practices) had significantly lower FoodProK scores compared to those following no dietary practices.

The observed associations remained consistent across all covariates in the sensitivity analysis using the modified FoodProK score (data not shown).

### 3.3 Association Between Self‐Reported HSR Understanding and Sociodemographic and Diet‐Related Characteristics

Results from the logistic regression analysis outlining the association between self‐reported understanding of HSR and sociodemographic and diet‐related characteristics are presented in Table 6. Respondents who identified as Aboriginal and/or Torres Strait Islander, those with missing BMI or aged ≥ 60 years had significantly lower odds of reporting the HSR was easy/very easy to understand, compared to Non‐Indigenous people, those with a BMI between 18.5–24.9 kg/m^2^ or aged 18–29 years, respectively.

**TABLE 6:** Association between self‐reported understanding of Health Star Rating (HSR) labelling and sociodemographic/diet‐related characteristics.

Variable	Odds ratio	95% CI	*p*
Sex at birth			
Male	Reference		
Female	1.11	0.95, 1.30	0.193
Age group (years)			
18–29	Reference		
30–44	0.96	0.75, 1.24	0.760
45–59	0.85	0.66, 1.10	0.218
≥ 60	**0.66**	**0.52, 0.85**	**0.001**
Education level			
Low	Reference		
Medium	0.96	0.80, 1.15	0.627
High	1.07	0.87, 1.32	0.517
Perceived income adequacy			
Very difficult/difficult	Reference		
Neither	1.07	0.87, 1.32	0.526
Easy/very easy	**1.47**	**1.19, 1.83**	**< 0.001**
Language spoken at home			
Only English	Reference		
Other language(s)	1.13	0.89, 1.42	0.319
Indigeneity			
Non‐Indigenous	Reference		
Aboriginal and/or Torres Strait Islander	**0.55**	**0.39, 0.78**	**0.001**
Body Mass Index (kg/m^2^)			
18.5–24.9	Reference		
< 18.5	0.94	0.59, 1.49	0.782
25.0–29.9	0.88	0.72, 1.08	0.237
≥ 30.0	0.88	0.71, 1.10	0.266
Missing	**0.74**	**0.58, 0.95**	**0.018**
Food shopping role			
Most	Reference		
Share equally	0.86	0.71, 1.04	0.114
Some, but not as much as others	0.98	0.70, 1.37	0.918
None	0.73	0.38, 1.38	0.330
Self‐reported nutrition knowledge			
Not at all/a little knowledgeable	Reference		
Somewhat knowledgeable	1.05	0.88, 1.24	0.604
Very/extremely knowledgeable	**1.66**	**1.30, 2.11**	**< 0.001**
Self‐reported healthiness of diet			
Poor/fair	Reference		
Good	1.00	0.83, 1.20	0.997
Very good/excellent	**1.36**	**1.08, 1.72**	**0.009**
Dietary restrictions			
No	Reference		
One or more (i.e., vegetarian, vegan, pescatarian, religious practices)	0.94	0.75, 1.19	0.628

*Note:* International Food Policy Study, Australia, 2021 (*n* = 3645). In bold: Statistically significant (*p* < 0.05).

Those who reported being very/extremely knowledgeable about nutrition, described the healthiness of their diets as very good/excellent or reported the greatest perceived income adequacy had higher odds of reporting that the HSR was easy/very easy to understand compared to those who reported being not at all/a little bit knowledgeable, having a poor/fair diet or the lowest perceived income adequacy. There were no statistically significant associations observed between understanding of HSR and sex, education level, language spoken at home, food shopping role or dietary restrictions.

## 4 Discussion

Overall self‐reported understanding of the HSR was high, with close to two thirds of respondents indicating they found the label ‘easy’ or ‘very easy’ to understand. These results are consistent with previous research indicating that the HSR graphic is easy to understand and use [31], with a five‐year review published in 2019 showing that 77% of Australians considered the HSR easy to understand [32]. Findings from a subsequent 2025 HSR monitoring report showed that most participants in Australia (around 90%) self‐reported at least some knowledge of the HSR [23]. Furthermore, objective measures of HSR understanding showed that the HSR‐only label format (compared with formats displaying additional nutrient information alongside the star rating) used in the present study was the easiest to interpret, with 85%–92% of participants correctly recognising that a higher HSR indicates a healthier product [23]. These findings indicate that self‐reported and objective understanding of the HSR are broadly aligned. Being able to easily understand FOPNLs is a key measure of their effectiveness as a food policy aimed at improving dietary patterns [31]. These findings demonstrate that HSR is perceived to be easily understood by most Australians, adding to the existing body of evidence on the performance of the HSR [23, 31, 32].

Moreover, our findings showed that respondents in Australia had a mean total FoodProK score of 4.8, which is relatively consistent with findings from a previous multi‐country analysis of the 2018 IFPS survey, where Australian adults had a FoodProK score of 5.0 based on similar food categories [19]. The product category scores were also consistent with the 2018 data analyses, with the lowest scores observed in the dairy category [28]. Sensitivity analysis showed that modified FoodProK scores (based on the healthiness ratings of unprocessed/minimally processed and UPF products only) were higher for all categories individually and overall compared with the original FoodProK scores, with a mean total modified FoodProK score of 6.0 (higher score indicating a higher alignment between perceived food healthiness and processing level). The findings indicate that more than half of respondents were able to correctly identify the healthiness of each of the assessed product categories in accordance with the lowest and highest levels of food processing, suggesting that participants' healthiness perceptions broadly reflected differences in food processing level. In addition, there was a positive, moderate association between the FoodProK scores and self‐reported HSR understanding. This indicates that respondents who found the HSR easier to understand generally scored higher in the assessment of food healthiness based on food processing level, suggesting that these are distinct yet complementary nutritional dimensions.

Findings from the FoodProK scores suggest that the intermediate processing food category (NOVA group 3) may introduce ambiguity, with higher FoodProK scores observed when it is excluded (modified FoodProK), revealing that participants find it easier to distinguish relative healthiness between unprocessed and UPF items. However, the same food categories tended to score lower in both FoodProK scores, suggesting that there was no substantial change in participants' overall pattern of interpretation across food categories. The modified FoodProK scores may have been higher than the original scores in part because the inaccurate classification of certain products (cheese block, cereal) could have made it more difficult to compare the relative healthiness of ultra‐processed versus processed products, and/or because respondents may have a better understanding of the relative healthiness of UPF compared to unprocessed/minimally processed products. Our results align with previous research in other countries showing that individuals are frequently able to correctly rate product healthiness according to level of processing and particularly identify UPFs as unhealthy foods [20]. A recent qualitative study from Australia found that participants had difficulty distinguishing processed foods from UPF [21], while qualitative research in the UK reported that food processing was commonly conceptualised as a continuum, with particular confusion surrounding the boundary between processed and UPF [33]. Our findings also align with a newly published Australian study in which respondents were asked to identify UPFs from 20 product images and 45% of UPFs were correctly identified, with chicken nuggets, fast‐food burgers, and instant noodles being the most frequently correctly identified UPFs [22]. Taken together, these findings support the use of the FoodProK score without the intermediate food processing category, which respondents appeared to find more ambiguous.

Differences in FoodProK scores across sociodemographic, BMI and diet‐related variables were observed. Significantly lower FoodProK scores were observed among respondents who had missing BMI, identified as Aboriginal and/or Torres Strait Islander, did none of the food shopping or had dietary restrictions. On the other hand, respondents with higher levels of self‐reported nutrition knowledge, females and older adults had significantly higher FoodProK scores. Previous research using the FoodProK scores from 2018 IFPS data similarly observed significantly lower scores in respondents with missing BMI data and those following restrictive dietary practices, while significantly higher scores were observed in older age groups, females and respondents with higher self‐reported nutrition knowledge [28]. This is consistent with research from Australia suggesting that men, younger age groups, and Aboriginal and/or Torres Strait Islander Peoples have lower food processing knowledge [22]. Across the literature, it has been consistently demonstrated that females and older age groups generally have higher levels of nutritional knowledge compared to men and younger age groups [18, 19, 34]. Similarly, sociodemographic differences were observed in terms of self‐reported ease of understanding the HSR. Respondents who reported higher perceived income adequacy, higher levels of nutrition knowledge or greater healthiness of their diet tended to report that the HSR was easy to understand, which is consistent with previous findings that show these groups have greater understanding of FOPNL [27]. Conversely, respondents within the older age group category (vs. 18–29 years), missing BMI (vs. 18.5–24.9 kg/m^2^) and those who identified as Aboriginal and/or Torres Strait Islander (vs. Non‐Indigenous) tended to have a lower reported understanding of HSR. Evidence from a HSR monitoring report also revealed disparities in the perceived knowledge across population groups [23]. Unlike our findings, the report found that Aboriginal and Torres Strait Islander participants reported higher self‐reported knowledge of the HSR; however, they were more likely to incorrectly believe that the HSR could be used to compare dissimilar food products. Furthermore, consistent with our findings, participants from lower socioeconomic status reported lower self‐rated HSR knowledge [23]. Notably, older respondents (≥ 60 years) scored higher on the FoodProK but reported lower understanding of HSR. This pattern aligns with previous studies indicating that nutrition label use tends to be lower among older adults [35], and that awareness and use of FOPNL are less common than use of nutrition information panels [27, 36]. Nutrition facts tables provide more detailed information about specific nutrients that older respondents may be conscious of as they are likely to have more health concerns than younger individuals, which could also mean they are more aware and cautious about the harms associated with industrial food processing [12].

Respondents following specific dietary practices like vegetarianism and veganism had lower FoodProK scores. This could be due to their use of a different set of criteria when assessing product healthiness (i.e., may broadly rate animal‐based products as less healthy) [35]. Lack of familiarity in judging and selecting food products based on their healthiness might also explain why people who do none of the food shopping scored significantly lower on the FoodProK. Finally, respondents with missing BMI data (extreme values, ‘don't know’ and ‘refuse to answer’ responses) had significantly lower FoodProK scores and lower self‐reported understanding of HSR. Although respondents with non‐normative weight might be more likely to avoid reporting their weight due to weight status or weight stigma [37], it is unclear how this would be connected to lower food processing knowledge. However, missing BMI data might be linked to lower engagement or attentiveness among respondents, which could have led to responses of lower quality.

Noteworthy, respondents who identified as Aboriginal and/or Torres Strait Islander had both lower FoodProK scores and lower self‐reported understanding of HSR. A likely contributing factor for these observations may be related to the economic and social marginalisation experienced by Aboriginal and Torres Strait Islander Peoples compared to Non‐Indigenous people [38]. Inequity gaps in literacy and numeracy skills are likely to extend to health literacy as well, although there is no nationally representative data on health literacy levels among Aboriginal and Torres Strait Islander Peoples [39]. Our findings highlight the importance of developing culturally appropriate food policy strategies and communication initiatives tailored to Aboriginal and Torres Strait Islander Peoples [40], with a clear need for further research on health and nutrition literacy and strategies for improving it in this population. A successful example of health literacy being improved among Aboriginal and Torres Strait Islander communities was the FOODcents program (replaced by Food Sensations for Adults) in Western Australia, where nutritional knowledge and fruit and vegetable intake improved following completion of a nutritional educational program developed to meet the specific knowledge and skill needs of the community [41]. More recent evidence from evaluations of the Food Sensations program, designed to improve participants' skills in planning, selecting, preparing, and consuming healthy foods, has also demonstrated positive effects on food literacy [42]. Grounding contemporary nutrition education in traditional food knowledge and practices can provide a meaningful approach to better support Aboriginal and Torres Strait Islander communities experiencing literacy and health inequities [43]. Future nutrition programs designed to enhance HSR understanding and food processing knowledge may also be strengthened through participatory approaches like co‐design to ensure that initiatives are culturally appropriate and sustainable over time.

Critically, the HSR label in Australia and New Zealand remains voluntary for manufacturers to display. After nearly 10 years of voluntary implementation, HSR uptake reached a maximum of 42% in 2021 and decreased to 39% in 2023 [44]. Following the final voluntary uptake period, the HSR was still displayed on only 39% of intended products in Australia by November 2025 [45], substantially below the 70% uptake target. Voluntary FOPNL schemes lead to uneven exposure, with application of the HSR skewed towards healthier products [27, 44]. Mandatory FOPNL implementation, which is being considered through a public consultation process in Australia and New Zealand [17], is likely to contribute to improving HSR understanding and mitigating inequities across population subgroups. Alongside mandatory adoption, incorporating food processing level into the HSR nutrient profiling algorithm to avoid UPFs receiving a favourable rating and developing tailored communication strategies could help improve understanding of both nutrient and food processing dimensions [16]. Qualitative research shows customers often rely on packaging cues to infer processing levels, supporting the integration of processing information into existing labelling systems [21].

One of the main strengths of this study is the large national sample used. Although recruitment was conducted using probability and non‐probability sampling methods, post‐stratification sample weights were applied based on sex, age group, education level and state/territory of residence to ensure the analytic sample was as representative of the Australian adult population as possible. In addition, this study used self‐reported measures (e.g., understanding of HSR was self‐reported but not objectively tested), which are effective for capturing data from large samples with minimal costs and researcher input; however, they are vulnerable to bias associated with social desirability, misreporting, and misunderstanding of questions. Nevertheless, even though the two assessed measures capture distinct dimensions of nutrition‐related knowledge, our analysis found a moderate, positive association between self‐reported HSR understanding and objective knowledge of food healthiness. Similarly, other analyses using IFPS data have shown that self‐reported understanding of nutrition labels is associated with objective measures of nutrition fact tables understanding [18].

Several limitations should also be acknowledged. One of the key limitations involved challenges applying NOVA classification criteria to specific food items and selecting products for use in the FoodProK tool that represented group 3 (processed) food items. Products were initially classified based on the general definitions and illustrative examples provided by the NOVA classification system, but subsequent further examination of specific ingredient lists affected the classification of several products [12]. For example, when designing the tool, the cheese block and cereals were believed to represent processed foods but subsequently, nutrition specialists determined that they should instead be classified as an ultra‐processed food based on their ingredients (some cheese and cereal products can be considered ‘processed’, but the cheese block shown contained modified milk ingredients and the cereal contained modified corn starch and an additive and thus were considered ‘ultra‐processed’). This means that two of the three items within the dairy and grain categories had overlapping NOVA classifications, which creates challenges in assessing respondent's ability to correctly classify food healthiness according to the level of processing through the FoodProK score. In recognition of these challenges, future IFPS survey measures related to food processing knowledge have been modified to focus solely on products that represent unprocessed/minimally processed foods (group 1) and UPF (group 4), and we conducted a sensitivity analysis in line with that approach. This approach also aligns with the focus of epidemiological studies comparing the health impact of dietary patterns high in UPF to diets primarily based on whole and minimally processed foods [3, 13].

Another important limitation of this study relates to the FoodProK score and its ability to capture knowledge of food processing. The survey measure used to calculate the FoodProK score did not explicitly ask respondents to assess food healthiness according to level of processing and, given that food items were shown together with nutrition facts tables and ingredients lists, it is uncertain whether participants were rating the product based on the perceived level of food processing, nutritional characteristics, a combination of both, or other aspects (e.g., vegetarian/vegan, food allergies, environmental impact). Evidence from Australia suggests that ingredient lists play an important role in food selection, with consumers commonly using them to identify healthier options [21]. Moreover, nutrition profiles and level of processing/NOVA classification might not always align (e.g., a food item could have higher levels of fat but be classified as healthier/less processed). However, the food products used in our study were generally aligned across these nutritional characteristics. Future research is warranted to further investigate food processing knowledge and understanding among the public, including further validation and refinement of tools to assess this dimension of food healthiness.

## 5 Conclusion

This study found inequities among Australian adults in both self‐reported understanding of HSR labels and the ability to assess food healthiness based on food processing level. These inequities across sociodemographic and diet‐related characteristics suggest that, even if mandated, the HSR may require tailored communication initiatives to improve understanding of nutrient‐based labelling and food processing and help ensure an equitable impact of FOPNL policies and dietary guidelines on supporting healthier food choices.

## Author Contributions

Conceptualization: C.G.‐D., C.M.W., D.H. Methodology: C.G.‐D., C.M.W., D.H. Formal analysis: J.P., C.G.‐D. Writing – original draft preparation: J.P., C.G.‐D. Writing – review and editing: T.S., A.J.C., C.M.W., D.H. Funding acquisition: C.G.‐D., A.J.C., D.H. Supervision: C.G.‐D., A.J.C., T.S. All authors have read and agreed to the published version of the manuscript.

## Funding

C.G.‐D was funded by Fundación Alfonso Martín Escudero and an Open University of Catalonia Postdoctoral Research Fellowship. C.G.‐D, T.S. and A.J.C. are researchers within the National Health and Medical Research Council (NHMRC) funded Centre of Research Excellence in Food Retail Environments for Health: Next Generation (RE‐FRESH: Next Generation) (APP2024716). The opinions, analysis, and conclusions in this paper are those of the authors and should not be attributed to the NHMRC. A.J.C. is a recipient of a Heart Foundation Future Leader Fellowship from the National Heart Foundation of Australia (Grant Number 102611). Funding for the International Food Policy Study (IFPS) was provided by a Canadian Institutes of Health Research (CIHR) Project Grant (PJT‐162167). The content is solely the responsibility of the authors and does not necessarily represent the official views of the Canadian Institutes for Health Research, the National Institutes of Health or other sources of funding. The funding agencies had no role in the design of the study, the collection, analysis, or interpretation of data, or in the writing or decision to submit the manuscript for publication.

## Conflicts of Interest

D.H. has provided paid expert testimony on behalf of public health authorities in response to challenges from the food and beverage industry. Other authors declare no conflicts of interest.

## Supporting information


**Table S1:** Mean healthiness rating for each food product.

## Data Availability

The data that support the findings of this study are available from the corresponding author upon reasonable request.
