# Supplementary material for: Food Processing Knowledge in Relation to Healthiness and Self‐Reported Understanding of the Health Star Rating in Australia
Source: Health Promot J Austr. 2026 Sep 3;37(4):e70235. doi: 10.1002/hpja.70235 (PMC13539369; doi:10.1002/hpja.70235)
Supplement: Supplementary file 1 [file HPJA-37-0-s001.docx]

**Supplementary Table 1.** Mean healthiness rating for each food product.

| **Assigned food processing level classification** | **Mean healthiness rating (SD)^*^** | | | |
| --- | --- | --- | --- | --- |
|  | **Grain** | **Dairy** | **Chicken** | **Fish** |
| **Group 1: Unprocessed & minimally processed** | **Oats** | **Milk** | **Chicken breast** | **Fish fillets** |
|  | 6.9 (8.3) | 4.4 (12.2) | 7.9 (1.8) | 7.8 (1.9) |
| **Group 3:  Processed**^†^ | **Cereal** | **Cheese block** | **Deli meat** | **Canned fish** |
|  | 3.2 (12.0) | 4.0 (10.5) | 5.8 (2.4) | 7.2 (2.1) |
| **Group 4:**  **Ultra-processed** | **Cereal bars** | **Cheese slices** | **Chicken nuggets** | **Battered fish fillets** |
|  | 2.6 (10.9) | 3.0 (11.8) | 3.9 (2.4) | 5.0 (2.3) |

^*^On a scale from 0 (not at all healthy) to 10 (extremely healthy)
^†^The cereal and cheese block products contained ingredient(s) characteristic of ultra-processed products.
